# Granzyme B^+^ CD4 T cells accumulate in the colon during chronic HIV-1 infection

**DOI:** 10.1080/19490976.2022.2045852

**Published:** 2022-03-08

**Authors:** Stephanie M. Dillon, Kaylee L. Mickens, Tezha A. Thompson, Emily H. Cooper, Sabrina Nesladek, Allison J. Christians, Moriah Castleman, Kejun Guo, Cheyret Wood, Daniel N. Frank, Katerina Kechris, Mario L. Santiago, Cara C. Wilson

**Affiliations:** a Department of Medicine, University of Colorado School of Medicine, Aurora, CO, USA; b Department of Biostatistics and Informatics, Colorado School of Public Health, Aurora, CO, USA

**Keywords:** HIV, gut, CD4 T cells, dysbiosis, granzyme B

## Abstract

Chronic HIV-1 infection results in the sustained disruption of gut homeostasis culminating in alterations in microbial communities (dysbiosis) and increased microbial translocation. Major questions remain on how interactions between translocating microbes and gut immune cells impact HIV-1-associated gut pathogenesis. We previously reported that *in vitro* exposure of human gut cells to enteric commensal bacteria upregulated the serine protease and cytotoxic marker Granzyme B (GZB) in CD4 T cells, and GZB expression was further increased in HIV-1-infected CD4 T cells. To determine if these *in vitro* findings extend *in vivo*, we evaluated the frequencies of GZB^+^ CD4 T cells in colon biopsies and peripheral blood of untreated, chronically infected people with HIV-1 (PWH). Colon and blood GZB^+^ CD4 T cells were found at significantly higher frequencies in PWH. Colon, but not blood, GZB^+^ CD4 T cell frequencies were associated with gut and systemic T cell activation and *Prevotella* species abundance. *In vitro*, commensal bacteria upregulated GZB more readily in gut versus blood or tonsil-derived CD4 T cells, particularly in inflammatory T helper 17 cells. Bacteria-induced GZB expression in gut CD4 T cells required the presence of accessory cells, the IL-2 pathway and in part, MHC Class II. Overall, we demonstrate that GZB^+^ CD4 T cells are prevalent in the colon during chronic HIV-1 infection and may emerge following interactions with translocated bacteria in an IL-2 and MHC Class II-dependent manner. Associations between GZB^+^ CD4 T cells, dysbiosis and T cell activation suggest that GZB^+^ CD4 T cells may contribute to gut HIV-1 pathogenesis.

## Introduction

Chronic immune activation and inflammation are major pathogenic features of HIV-1 infection. These features have been linked to the sustained disruption of intestinal mucosal homeostasis which persists even in the presence of effective viral suppression with antiretroviral therapy (ART).^1,2^ Early in infection, HIV-1 replicates to high levels in intestinal CD4 T cells, resulting in the massive depletion of T helper (Th) 17 and Th22 subsets that play critical roles in maintaining intestinal homeostasis.^3^ Disruption of gut homeostasis leads to mucosal inflammation and epithelial barrier damage, culminating in increased microbial translocation of enteric bacteria and their inflammatory products into the underlying tissue and systemic circulation.^4^ Furthermore, numerous studies, including our own, have demonstrated an altered intestinal microbiome (dysbiosis) among people with HIV-1 (PWH), with dysbiotic profiles correlating with local and systemic immune activation, inflammation and microbial translocation (reviewed in^5^), metabolic syndrome,^6^ and various inflammation-associated comorbidities.^7^

To understand the crosstalk between translocating dysbiotic enteric bacteria and the human gut immune system, we and others have utilized cell culture models with primary human intestinal lamina propria mononuclear cells (LPMC). Data obtained from these *ex vivo* models highlighted that enteric pathogenic and commensal bacteria-reactive CD4 T cells are normal components of the gut CD4 T cell repertoire.^8–11^ Furthermore, commensal enteric bacteria, including species of Gram-negative bacteria that are increased in abundance in PWH, drive LP CD4 T cell activation and proliferation and increase productive HIV infection and CD4 T cell death *in vitro*.^9,12–14^ To probe pathways that may be involved in gut mucosal HIV-1 pathogenesis, we recently performed unbiased transcriptomics to profile LP CD4 T cell gene expression following *in vitro* exposure to enteric commensal *Prevotella stercorea* in the presence or absence of experimental HIV-1 infection.^15^ We observed that exposure of LPMC to enteric bacteria induced significant Granzyme A (GZA) and GZB expression in gut CD4 T cells, and the expression of these granzymes was further enhanced in the presence of replicating HIV-1. Granzymes are serine proteases, best known as markers of cytotoxic CD8  T cells and natural killer cells, and are not commonly associated with the ‘helper’ function of CD4 T cells. Mechanistically, granzymes initiate apoptosis and cell death in target cells in conjunction with perforin-mediated trafficking.^16^ In addition, granzymes could promote inflammatory cytokine release, potentiate cytokine responses initiated by bacterial lipopolysaccharide (LPS) or Gram-negative bacteria, promote extracellular matrix remodeling, and directly kill bacteria.^17–20^ Thus, our *in vitro* observations suggested that translocating dysbiotic bacteria in PWH could lead to high expression of GZB in gut CD4 T cells. Induction of these potentially highly inflammatory cells may then contribute to gut-associated HIV-1-pathogenesis.

Several studies have detailed the emergence of CD4 T cells with cytolytic activity and HIV-1-specificity in the blood during HIV infection.^21–33^ Frequencies of cytotoxic CD4 T cells (CD4 CTLs), variably identified by granzyme type (i.e. GZA, GZB, GZK) and co-expression of other cytolytic molecules such as perforin and/or degranulation molecules (e.g. CD107a), increased early during HIV-1 infection. CD4 CTLs were present throughout disease, remained detectable in the presence of ART and had cytolytic activity against HIV-1-infected cells. GZA-expressing blood CD4 T cell frequencies were linked to lower viral set point, suggesting a host-protective role.^23,30^ Notably, lymph nodes of PWH with or without ART contained few CD4 CTLs, contrasting with the higher frequencies observed in blood.^33^ This finding suggested that CD4 CTL-mediated control of HIV-1 may be compartmentalized *in vivo*. However, few studies have directly investigated the presence of CD4 CTLs in human gut tissue from either PWH or uninfected persons. A CD4 CTL phenotype was induced in blood CCR5^+^ Th1 and Th17 cells *in vitro*^34^ suggesting a similar phenotype may be observed in the gut given the preponderance of these Th subsets in this tissue site. One study showed that nonhuman primate species that support pathogenic SIV infection (uninfected rhesus and pigtail macaques) harbored more GZB^+^ CD4 T cells in the colon compared to nonpathogenic SIV hosts (uninfected sooty mangabeys and African green monkeys).^35^ The authors postulated that GZB release from CD4 T cells may be a contributing factor to epithelial barrier disruption and contribute to SIV pathogenesis, but the impact of SIV infection on these cells was not investigated.

Dysbiotic microbiome profiles in PWH have been linked to mucosal and systemic inflammation and microbial translocation,^36^ and GZB expression in both uninfected and HIV-1-infected LP CD4 T cells was driven by exposure to commensal bacteria *in vitro*.^15^ Thus, we hypothesized that GZB expression in gut CD4 T cells of PWH would be elevated and linked to dysbiosis and to local and systemic features of HIV-1 pathogenesis. To test this hypothesis, we evaluated the frequencies of GZB^+^ CD4 T cells in archived colonic biopsies from untreated PWH and uninfected controls from a completed clinical study^36,37^ and explored *in vivo* relationships with markers of mucosal and systemic inflammation. To determine if GZB expression was specific to gut CD4 T cells, we similarly evaluated GZB expression in study participant-matched blood CD4 T cells. To probe potential pathways linking induction of GZB-expressing cells to translocating enteric commensal bacteria, we used a human LPMC *in vitro* model^38^ to gain mechanistic insights on how commensal bacteria upregulated GZB in human gut CD4 T cells.

## Results

### Clinical study participant characteristics and study design

**Figure 1.**
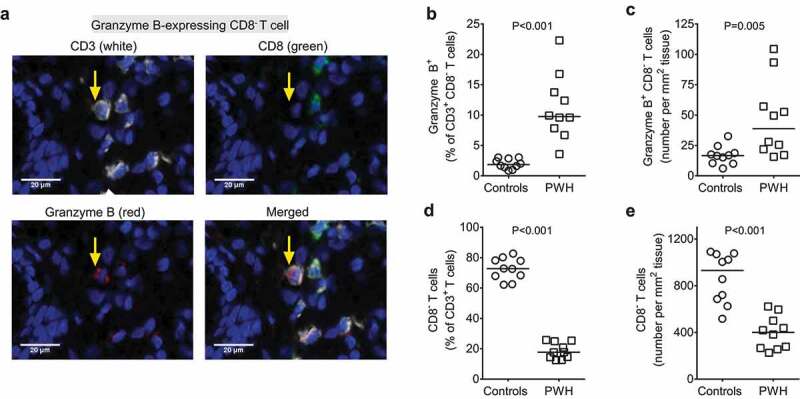
**People with HIV have higher frequencies of colonic Granzyme B-expressing lamina propria CD4 T cells versus controls**. Colonic formalin-fixed paraffin-embedded (FFPE) tissue sections were obtained from uninfected controls (*N* = 10) and from people with HIV (PWH; *N* = 10) who were not receiving anti-retroviral therapy and frequencies of lamina propria (LP) granzyme B (GZB)-expressing T cells evaluated using immunofluorescence microscopy. (a) Representative colonic tissue section from a PWH stained with CD3 (white; top left panel), CD8 (green; top right panel), GZB (red; bottom left panel) and DAPI (blue). Due to down-regulation of CD4 by HIV,^39^ CD4 T cells were identified as CD8^neg^. The yellow arrow highlights the same cell identified as CD3^+^ CD8^−^ GZB^+^ (red) and merged (bottom right panel). Images are shown as 40x and a scale bar indicated. (b) Percentages and (c) number of GZB^+^ colonic LP CD4 (CD8^−^) T cells and (d) percentages and (e) number of colonic LP total CD4 (CD8^−^) T cells in controls (circles) and PWH (squares). Lines indicate median values. Statistical analysis: two-sample Mann-Whitney U tests.

GZB-expressing CD4 T cells were quantified in archived, formalin-fixed, paraffin-embedded (FFPE) colon LP tissue sections obtained from chronically infected PWH (*N* = 10) and uninfected controls (*N* = 10) by immunofluorescence. PWH were ART-naive or had not been on treatment for >7 days in the preceding 6 months (see Supplementary Methods for additional inclusion/exclusion details). The study participants were selected from a larger, previously completed clinical study that included 17 PWH and 14 uninfected controls with previously acquired colonic mucosa-associated bacterial microbiome datasets.^36,37^ Data on systemic and colonic indicators of inflammation and immune activation were also previously compiled for these cohorts.^36,37^ PWH were retrospectively selected for this current study based on blood CD4 counts ≥400 cells/μl to enrich for participants with an adequate number of colon CD4 T cells and permit accurate histological enumeration of GZB^+^ cells. To address our hypothesis that GZB expression would be linked to dysbiosis and to local and systemic features of HIV-1 pathogenesis, the final cohort of PWH was then selected based on the highest ranking of the following features of HIV-1 pathogenesis: (1) colonic tissue HIV-1 RNA levels (HIV-1 replication), (2) systemic IL-6 levels (inflammation) and (3) systemic LPS levels (microbial translocation). Uninfected controls with blood CD4 counts ≥400 cells/μL were selected to balance both age and sex of the PWH cohort. Study participant characteristics are detailed in Table S1. Due to downregulation of CD4 by HIV-1,^39^ CD4 T cells were identified as CD3^+^CD8^neg^ T cells (Figure 1a).

### Frequencies of LP GZB-expressing CD4 T cells are increased in colons of untreated, chronically-infected PWH

LP GZB^+^ CD4 T cells were present at low frequencies in the absence of HIV-1 infection (controls: median 1.8% of CD4 T cells, range: 0.37–3.0; Figure 1b). In contrast, LP GZB^+^ CD4 T cells were readily detected at significantly higher frequencies in PWH versus controls, both as a fraction of total CD4 T cells (PWH: 9.8%, 3.6–22.3; *P* < .001) (Figure 1b) and per area of LP tissue (PWH: 39/mm^2^, 16–104; Controls: 17/mm^2^, 6–33; *P* = .005) (Figure 1c). As expected, percentages of LP CD4 T cells as a fraction of total LP CD3^+^ T cells, and as the number of CD4 T cells per area of LP tissue, were significantly lower in PWH consistent with gut CD4 T cell depletion^3^ (PWH: 17.7%, 12.5–25.7 versus Controls: 72.7%, 62.2–82.7, *P* < .001; PWH: 400/mm^2^, 226–622 versus Controls: 930/mm^2^, 517–1090, *P* < .001) (Figure 1d, e).

γδ T cells can express GZB as well as CD4 and CD8, and small populations of γδ T cells have been reported in human gut LP.^40–42^ Therefore, to confirm that LP GZB^+^ CD8^neg^ T cells measured in our study did not reflect γδ T cells, we performed a histological analysis of LP GZB^+^ γδ cells in a subset of study participants (*N* = 3 per group) using the same staining panel (CD3, CD8, GZB) and included an antibody directed against γδ T Cell Receptor (TCR). LP γδ TCR^+^ cells constituted a small fraction of total CD3^+^ T cells in both controls (mean ± SEM: 3.7 ± 1.1%) and PWH (2.7 ± 0.3%); the majority of LP γδ TCR^+^ cells did not express CD8 (controls: 78.8 ± 6.8%; PWH: 61.9 ± 7.5%) (Figure S1). Of these CD8^neg^ γδ TCR^+^ cells, less than 5% expressed GZB in both controls (3.7 ± 0.8%) and PWH (4.8 ± 0.5%). Importantly, LP GZB^+^ CD8^neg^ γδ TCR^+^ cells only accounted for a small fraction of total GZB^+^ CD8^neg^ T cells in both controls (10.9 ± 3.9%) and PWH (7.9 ± 4.6%). Thus, the majority of the accumulated GZB CD8^neg^ T cells in PWH were likely canonical CD4 T cells.

### Frequencies of LP GZB-expressing CD4 T cells associate with systemic and colonic T cell activation

To determine if the frequencies of LP GZB^+^ CD4 T cells correlated with features of HIV-1 pathogenesis, linear regression models adjusting for age, sex and HIV-1 status were used to determine associations between GZB^+^ CD4 T cells and various archived clinical, systemic and colon biopsy readouts.^36,37^ Variables were measured in at least 18 participants. HIV-associated differences in systemic and colonic readouts noted in the larger cohort were similarly observed in the smaller group (Figure S2).^36,37^ These included higher levels of blood T cell activation and biomarkers indicative of inflammation (IL-6, hs-CRP), immune activation (soluble CD14 (sCD14), sCD27) and microbial translocation (LPS, LTA) in PWH (Figure S2A). We additionally measured serum levels of both GZA and GZB. Notably, serum GZA, but not GZB levels were significantly higher in PWH (Figure S2A), profiles likewise reflected in the larger cohorts (GZA: PWH 64.8 pg/ml, 3.6–167, Controls: 18.3, 0–75.5, *P* < .0001; GZB: PWH 8.5, 0–18.4, Controls 6.5, 0–12.2, *P* = .38) (Figure S3). Within colonic tissue, frequencies of CD8 T cells were higher in PWH, whereas the frequencies of CD4 T cells and CD11c^+^ myeloid dendritic cells (mDCs) were similar between the groups (Figure S2B,D). Levels of colon T cell and mDC activation were higher in PWH (Figure S2C,E).

Frequencies of LP GZB^+^ CD4 T cells, enumerated as the number per area of tissue and as a percentage of total LP CD4 T cells, significantly associated with the percentage of blood CD4 and CD8 T cell co-expressing CD38 and HLA-DR (i.e., activated) (Table 1). Estimates provided by the modeling indicated that the number of LP GZB^+^ CD4 T cells increased by 16.3 for each 1% increase in the percentage of activated blood CD4 T cells and by 2.8 for activated blood CD8 T cells. Moreover, both the number and percentage of LP GZB^+^ CD4 T cells significantly associated with the number of colonic CD38^+^HLA-DR^+^ CD4 or CD8 T cells per gram of tissue (Table 1). Significant associations were also noted between LP CD4 GZB^+^ T cells and the number of colonic CD11c^+^ mDCs (Table S2). Percentages of LP GZB^+^ CD4 T cells did associate with the number of plasmacytoid DCs (pDCs), but this weak association (*P* = .04) was lost when adjusting for multiple tests. No significant associations (either with or without adjustment for multiple tests) were observed between LP GZB^+^ CD4 T cell frequencies and colonic CD4 and CD8 T cell frequencies, mDC activation or HIV-1 RNA levels (Table S2). Similarly, LP GZB^+^ CD4 T cells did not significantly associate with blood CD4 T cell count, HIV-1 viral load (PWH only), systemic levels of inflammation (IL-6, hs-CRP, IFABP, sCD14, sCD27), granzymes (GZA, GZB) or microbial translocation markers (LPS, LTA) (Table S3).

**Table 1.** Associations between colonic granzyme B-expressing lamina propria CD4 T cells and systemic and colonic T cell activation

Predictor	Outcome: GZB^+^ CD4 T cells*	Estimate	Unadjusted *P* value	FDR value
*Peripheral blood T cell activation*				
CD38^+^ HLA-DR^+^ CD4 T cells^†^	Number	16.3	**0.002**	**0.02**
	Percentage	0.03	**0.0009**	**0.006**
CD38^+^HLA-DR^+^ CD8 T cells^†^	Number	2.76	**0.004**	**0.02**
	Percentage	0.004	**0.03**	0.13
*Colonic T cell activation*				
CD38^+^ HLA-DR^+^ CD4 T cells^‡^	Number	0.0002	**0.00002**	**0.0004**
	Percentage	0.0000003	**0.0007**	**0.006**
CD38^+^ HLA-DR^+^ CD8 T cells^‡^	Number	0.00006	**0.0001**	**0.002**
	Percentage	0.0000001	**0.0004**	**0.006**

*GZB-expressing lamina propria CD4 T cells were identified as CD3^+^ CD8^−^ and reported as either the number of GZB^+^ CD8^−^ T cells per tissue area (mm^2^) or as the percentage of total CD8^−^ T cells. Archived study participant-matched measurements of peripheral blood or colonic T cell activation were obtained from a previously completed clinical study.^36,37^ Multi-color flow cytometry was used to evaluate peripheral blood (PB) or colonic CD4 and CD8 T cells co-expressing CD38 and HLA-DR and were expressed as the ^†^percentage of PB CD4 or CD8 T cells or as the ^‡^number of colonic CD4 or CD8 T cells per gram of mucosa. Linear regression models were used to evaluate the association between predictor and each outcome in all study participants when adjusting for age, sex and HIV status. *P* values were adjusted for multiple tests using Benjamini-Hochberg False Discovery Rate (FDR). Bold *P* values indicate significant associations for the unadjusted *p*-value < 0.05 or for an alpha of 0.05 between predictor and outcome.

### Frequencies of LP GZB-expressing CD4 T cells associate with the relative abundance of Prevotella species

We previously reported that HIV-associated alterations in the colonic mucosa bacterial microbiome were linked to mucosal and systemic immune activation.^36^ To probe potential relationships between LP GZB^+^ CD4 T cells and the colonic mucosa-associated microbiota, we performed linear regression analysis (adjusting for age, sex and HIV status) using the same bacterial taxa (and relative abundance (RA) values) previously shown to be altered in abundance in the larger cohort of PWH relative to controls.^12,36,37,43^ Of note, differences in RA between controls and PWH for the specific taxa investigated were generally reflected in the smaller cohort studied here (Figure S4, Table S4). These included higher RA of Proteobacteria (e.g. *Acinetobacter junii*) and *Prevotella* species and decreased abundances of taxa belonging to Ruminococcaceae, Lachnospiraceae (e.g. Blautia, Coprococcus), Bacteroides and butyrate-producing species (i.e. summed RA of colonic bacterial species known to produce butyrate, and *Roseburia intestinalis*).

Significant positive associations between frequencies of LP GZB^+^ CD4 T cells and the RA of specific *Prevotella* species, and the RA of multiple taxa to which those species belong (i.e. Phylum: Bacteroidetes, Family: Prevotellaceae and Genus: *Prevotella*) were observed (Table 2). For example, each 1% increase in RA of *P. stercorea* was associated with an estimated increase of 249.3 LP GZB^+^ CD4 T cells and each 1% increase in *P. copri* with an estimated increase of 79.5 LP GZB^+^ CD4 T cells. The association between the percentage of LP GZB^+^ CD4 T cells and *P. copri* RA did not reach statistical significance (unadjusted *P* value = .07). No statistically significant associations (either with or without adjustment for multiple tests), were observed between LP GZB^+^ CD4 T cell frequencies and RA of the other phyla, families, genera (Table S5) or species investigated (Table S6). In secondary analyses, multivariate linear regression models were adjusted for age, sex, HIV and MSM status (recorded as Yes/No) and similar relationships between LP GZB^+^ CD4 T cells frequencies and RA of *Prevotella* species (and higher taxa) were observed (data not shown).

**Table 2.** Associations between colonic granzyme B-expressing lamina propria CD4 T cells and relative abundance of colonic mucosa-associated *Prevotella* species

Predictor [Prevalence^¥^]	Outcome: GZB^+^ CD4 T cells*	Estimate	Unadjusted *P* value	FDR value
Phylum: Bacteroidetes^†^	Number	71.1	**0.002**	**0.02**
[100%]	Percentage	0.101	**0.01**	0.16
Family: Prevotellaceae^*†^	Number	69.9	**0.0009**	**0.01**
[100%]	Percentage	0.091	**0.02**	0.16
Genus: Prevotella^*†^	Number	76.4	**0.0004**	**0.01**
[100%]	Percentage	0.099	**0.01**	0.16
Prevotella stercorea^*‡^	Number	249.3	**0.005**	**0.03**
[80%]	Percentage	0.470	**0.001**	**0.03**
Prevotella copri^*‡^	Number	79.5	**0.003**	**0.02**
[95%]	Percentage	0.09	0.07	0.43

*GZB-expressing CD4 T cells were identified as CD3^+^ CD8^−^ and reported as either the number of GZB^+^ CD8^−^ T cells per tissue area (mm^2^) or as the percentage of total CD8^−^ T cells. Archived study participant-matched measurements of colonic mucosa-associated bacterial taxa were obtained from a previously completed clinical study.^36,37^ Units: ^†^relative abundance as percentage of total bacteria or as ^‡^percentage of classified species. ^¥^Prevalence of each taxa across the study cohort (% of study participants with detectable taxa). Linear regression models were used to evaluate the association between predictor and each outcome in in all study participants when adjusting for age, sex and HIV status. P values were adjusted for multiple tests using Benjamini-Hochberg False Discovery Rate (FDR). Bold *P* values indicate significant associations for the unadjusted *p*-value < 0.05 or for an alpha of 0.05 between parameter and outcome.

### Frequencies of peripheral blood GZB^+^ CD4 T cells are higher in PWH, but do not associate with measured features of HIV-1 pathogenesis

**Figure 2.**
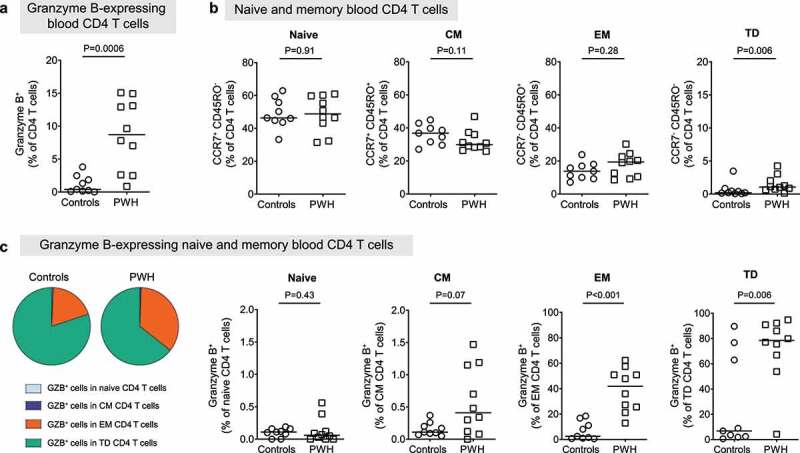
**People with HIV have higher frequencies of blood granzyme B-expressing CD4 T cells**. PBMC samples were obtained from uninfected controls (circles; *N* = 9) and people with HIV (PWH) (squares, *N* = 10) who were not receiving anti-retroviral therapy. (a) Percentages of granzyme B (GZB)-expressing CD4 T cells. (b) Percentages of naïve (CCR7^+^CD45RO^−^), central memory (CM; CCR7^+^CD45RO^+^), effector memory (EM; CCR7^−^CD45RO^+^) and terminally-differentiated effector memory (TD; CCR7^−^CD45RO^−^) CD4 T cells. (c) Pie-chart illustrating the average percentage of GZB-expressing cells in naïve, CM, EM or TD CD4 T cells in controls and PWH. (d) Percentages of GZB^+^ CD4 T cells naïve, CM, EM and TD T cell subsets in controls and PWH. Lines indicate median values. Statistical analysis: two-sample Mann-Whitney U tests.

To determine if the increased frequencies of LP GZB^+^ CD4 T cells in PWH were unique to the colon or were also observed systemically, GZB^+^ CD4 T cells were enumerated in study participant-matched peripheral blood (PB) using multicolor flow cytometry (Figure S5). Similar to colon tissue, the percentages of PB CD4 T cells expressing GZB (as a fraction of total CD4 T cells) were generally low in controls (0.4%, 0.01–3.8) and significantly higher in PWH (8.7%, 0.8–15.1; *P* = .0006) (Figure 2a). However, in contrast to LP GZB^+^ CD4 T cells, the percentages of PB GZB^+^ CD4 T cells did not significantly associate with any systemic or colon measurements (Table S7) or with microbiota RA (Table S8), when adjusting for age, sex and HIV-1 status and multiple testing. Similar to the percentage of LP GZB^+^ CD4 T cells (Table 2), the RA of *P. copri* was weakly associated with the percentage of PB GZB^+^ CD4 T cells (Table S8).

Given that the majority of colonic CD4 T cells are effector memory cells,^44^ we also evaluated the expression of GZB in memory and naïve PB CD4 T cell populations (Figure S5). Percentages of naïve (CCR7^+^CD45RO^−^), central memory (CCR7^+^CD45RO^+^; CM) and effector memory (CCR7^−^CD45RO^+^; EM) PB CD4 T cells were generally similar between controls and PWH (Figure 2b). Terminally differentiated memory (CCR7^−^CD45RO^−^; TD) CD4 T cells were significantly higher in PWH (*P* = .006) although constituted less than 2% of the total CD4 T cells. In both controls and PWH, GZB was preferentially expressed in EM and TD populations and percentages of GZB^+^ EM and TD CD4 T cells were significantly higher in PWH (Figure 2c).

Local reactivation of murine skin tissue-resident CD8^+^ T cells lead to their migration into the circulation (termed ‘ex-resident memory T cells’).^45^ To explore if the observed higher frequencies of PB GZB^+^ EM CD4 T cells in PWH potentially reflected LP GZB^+^ CD4 T cells migrating into the blood as a result of the ongoing gut immune activation, we regressed frequencies of PB GZB^+^ EM CD4 T cells on frequencies of LP GZB^+^ CD4 T cells in PWH only (*N* = 10, adjusted for age and sex). A significant positive relationship between the percentage of PB GZB^+^ EM CD4 T cells with the number of LP GZB^+^ CD4 T cells was noted (estimate 0.9227, *P* = .003).

### LP CD4 T cells upregulate GZB expression following exposure to a panel of enteric bacteria *in vitro*

Our *in vivo* findings suggest a significant relationship between LP GZB^+^ CD4 T cells, T cell activation and the colonic bacterial microbiome. Previously, we showed that *Prevotella* abundance in PWH is associated with colonic T cell activation.^36^ We subsequently utilized an *in vitro* human LPMC model (using healthy gut tissue) to demonstrate that LP CD4 T cells were not only activated in response to exposure to various *Prevotella* species,^12^ but exposure to *Prevotella stercorea* also stimulated high levels of GZB expression.^15^ We therefore utilized the same *in vitro* human LPMC model to dissect potential mechanisms driving microbe-induced GZB expression in human gut LP CD4 T cells. These experiments were performed in the absence of HIV-1 to avoid the confounding impact of HIV-1-associated LP CD4 T cell death.

We first evaluated GZB expression in LP CD4 and CD8 T cells measured directly *ex vivo* (baseline) using flow cytometry (Figure S6A). In keeping with the histological analysis of tissue in uninfected controls (Figure 1), a very low fraction of LP CD4 T cells expressed GZB at baseline (mean ± SEM: 0.09 ± 0.05% of CD4 T cells), whereas LP CD8 T cells constitutively expressed GZB to a greater degree (15.5 ± 6.5% of CD8 T cells) (Figure S6B).

**Figure 3.**
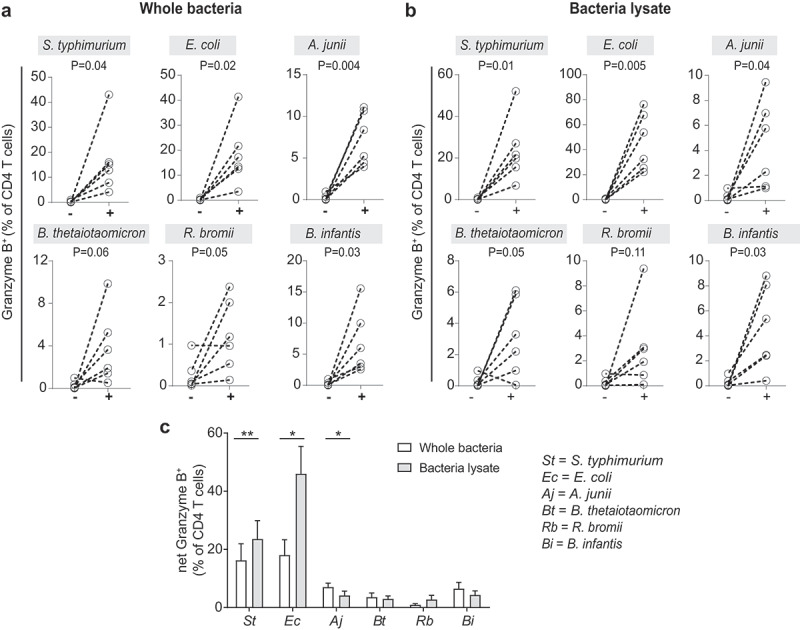
**Exposure of LPMC to enteric bacteria induce granzyme B expression in lamina propria CD4 T cells**. LPMC (*N* = 6) were exposed *in vitro* to a panel of (a) whole bacteria [2.5 bacteria:1 lamina propria mononuclear cell (LPMC); (+)] or (b) bacterial lysates [10 μg/ml; (+)] or were cultured without exogenous stimuli (-) for 4 days. (c) Percentages of granzyme B (GZB)^+^ LP CD4 T cells induced in response to *in vitro* exposure to whole bacteria or bacterial cell lysates were compared. Values are shown as percentage of GZB^+^ LP CD4 T cells in response to stimulation minus percentages detected in unstimulated conditions (net). Bars indicate mean+SEM. Statistical analysis: Paired *t*-tests; *P < .05, **P < .01.

To determine if other bacterial species (in addition to *P. stercorea*),^15^ could induce GZB expression in LP CD4 T cells, LPMC were similarly cultured in broad spectrum antibiotics for 4 days with a panel of commensal enteric bacteria (whole cell (WC) or lysates) (Figure S7). This panel including enteric species representing Gram-negative pathogenic (*Salmonella typhimurium*) and commensal (*Escherichia coli*) bacteria, a Gram-positive probiotic (*Bifidobacterium infantis*) and species previously shown to be higher (*A. junii, Bacteroides thetaiotaomicron*; both Gram-negative) or lower (*Ruminococcus bromii*; Gram-positive) in abundance in colonic tissue of PWH.^12,37^ *S. typhimurium* and *E. coli* induced the greatest percentages of GZB^+^ LP CD4 T cells above unstimulated conditions (*S. typhimurium* average fold increase WC 65×; lysate 93×; *E. coli* WC 72×; lysate 181×) (Figure 3). Other Gram-negative bacteria also increased GZB^+^ LP CD4 T cells, but to a lesser degree (*A. junii* WC 29×; lysate 17×; *B. thetaiotaomicron* WC 15×; lysate 12×). GZB expression was also noted in response to stimulation with Gram-positive bacteria (*B. infantis* WC 26×; lysate18x; *R. bromii* WC 5×; lysate 12×). These data demonstrate that diverse enteric commensal bacteria could drive GZB expression in gut CD4 T cells, although not to the same extent.

### GZB is induced rapidly in LP CD4 T cells in response to stimulation of LPMC with *E. coli* lysate

Given the robust GZB induction in response to *E. coli* (Figure 3c), further mechanistic studies on bacteria-induced GZB expression in human gut CD4 T cells were undertaken with *E. coli* lysate and measured using multi-color flow cytometry. To determine the kinetics of GZB induction, LPMCs were exposed to *E. coli* for 24 hours, 48 hours and 4 days (96 hours) and GZB expression evaluated. By 24 hours, variable levels of GZB expression were noted (range 2.5–38.9%) (Figure S8). At 48 hours, GZB expression levels increased a further 4.2× with values significantly higher than baseline (0 hours). A plateau was reached for GZB induction in LP CD4 T cells by 4 days, which was the time point used in downstream experiments.

### Commensal *E. coli* stimulation induces robust GZB expression in CD4 T cells from gut, but not blood or lymphoid tissue

**Figure 4.**
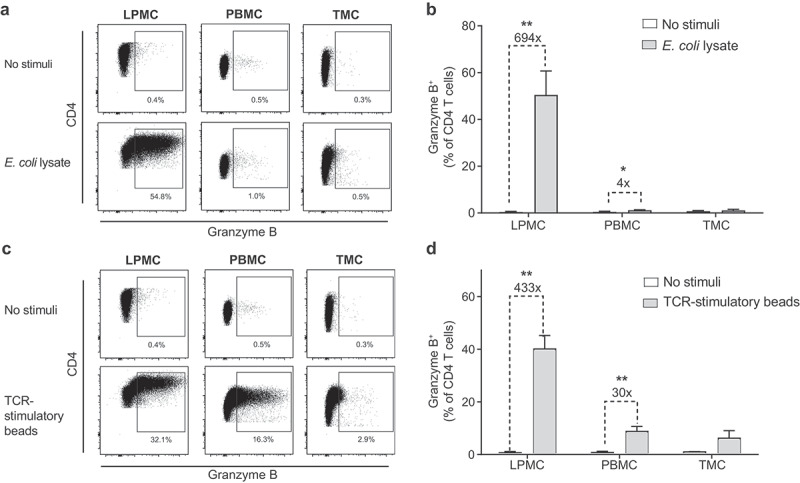
***E. coli* and T cell receptor-mediated stimulation induce substantial granzyme B expression in lamina propria but not in blood or tonsil CD4 T cells**. Lamina propria mononuclear cells (LPMC, *N* = 5), peripheral blood mononuclear cells (PBMC, *N* = 5) or tonsil mononuclear cells (TMC, *N* = 5) were exposed *in vitro* to *E. coli* (10 μg/ml) or T cell receptor (TCR)-stimulatory beads (1 bead: 25 LPMC or TMC; 1 TCR bead: 2 PBMC) or cultured without exogenous stimuli (no stimuli) for 4 days. Representative flow profiles of granzyme B (GZB) expression in CD4 T cells following stimulation with (a) *E. coli* or (c) TCR-stimulatory beads. LP CD4 T cells were identified in viable, single cell CD3^+^ lymphocytes. A matched isotype control was used to established GZB expression. Percentages of CD4^+^ T cells expressing GZB in response to exposure to (b) *E. coli* or (d)TCR-stimulatory beads with isotype values removed. Bars indicate mean+SEM. Statistical analysis: Paired *t*-test; *P < .05, **P < .01. (x) indicates average fold increase in percentage of GZB^+^ CD4 T cells in stimulated versus unstimulated conditions.

To determine if induction of GZB expression by enteric bacteria *in vitro* was a common feature among CD4 T cells from multiple sites or specific to those in gut tissue, PB mononuclear cells (PBMC) and disaggregated tonsil cells were similarly cultured with or without *E. coli* lysate for 4 days (Figure 4a). In contrast to the robust induction of GZB in LP CD4 T cells (693×), *E. coli* exposure of PBMC induced a significant increase of PB CD4 T cells expressing GZB, but the average increase above unstimulated PBMC was only 4-fold (Figure 4b). No significant increase in GZB expression in tonsil CD4 T cells was observed.

Bacteria-reactive CD4 T cells are less frequent in blood than gut tissue,^10^ which may explain the apparent site-specific differences in GZB expression in response to bacterial stimulation *in vitro*. Therefore, the capacity of LP, PB or tonsil CD4 T cells to express GZB in response to direct TCR stimulation using TCR-stimulatory beads (anti-CD3, CD2, CD28) was investigated (Figure 4c). TCR stimulation of PBMC significantly increased percentages of GZB^+^ PB CD4 T cells above no stimulation (30×), but remained significantly lower than GZB^+^ LP CD4 T cells (433×; *P* = .009) (Figure 4d). TCR stimulation did not significantly induce GZB in tonsil CD4 T cells.

### E. coli-induced GZB expression in LP CD4 T cells occurs indirectly and is partially mediated by HLA-DR

We previously demonstrated robust proliferation and increased frequencies of activated (CD38^+^ HLA-DR^+^) LP CD4 T cells following bacterial stimulation of gut LPMC *in vitro*.^9,11,12^ We also reported that bacteria-induced proliferation of LP CD4 T cells *in vitro* was dependent on antigen presenting cells (APC; e.g., mDCs) and was mediated in an MHC Class II-dependent manner.^9,11,12^ To determine the degree to which microbe-induced GZB expression tracked with proliferation and activation of LP CD4 T cells, LPMC were exposed to *E. coli* and fractions of proliferating or activated cells co-expressing GZB enumerated 4 days later (Figure S9). On average, 19.1 ± 2.4% of LP CD4 T cells proliferated (CFSE^lo^) in the presence of *E. coli* versus minimal proliferation in unstimulated conditions (0.5 ± 0.2%; *N* = 7). A majority of proliferated LP CD4 T cells expressed GZB (69.7 ± 7.2%) (Figure S9a). *E. coli* stimulation of LPMC also increased the percentages of LP CD4 T cells co-expressing CD38 and HLA-DR (10.5 ± 0.6%) above unstimulated LPMC (4.3 ± 1.6%; *N* = 3), with approximately half (54.7 ± 1.7%) of activated LP CD4 T cells expressing GZB (Figure S9b). Moreover, only 23.8% (± 5.9%; *N* = 4) of *E. coli*-induced GZB^+^ LP CD4 T cells were CD38^+^HLA-DR^+^.

**Figure 5.**
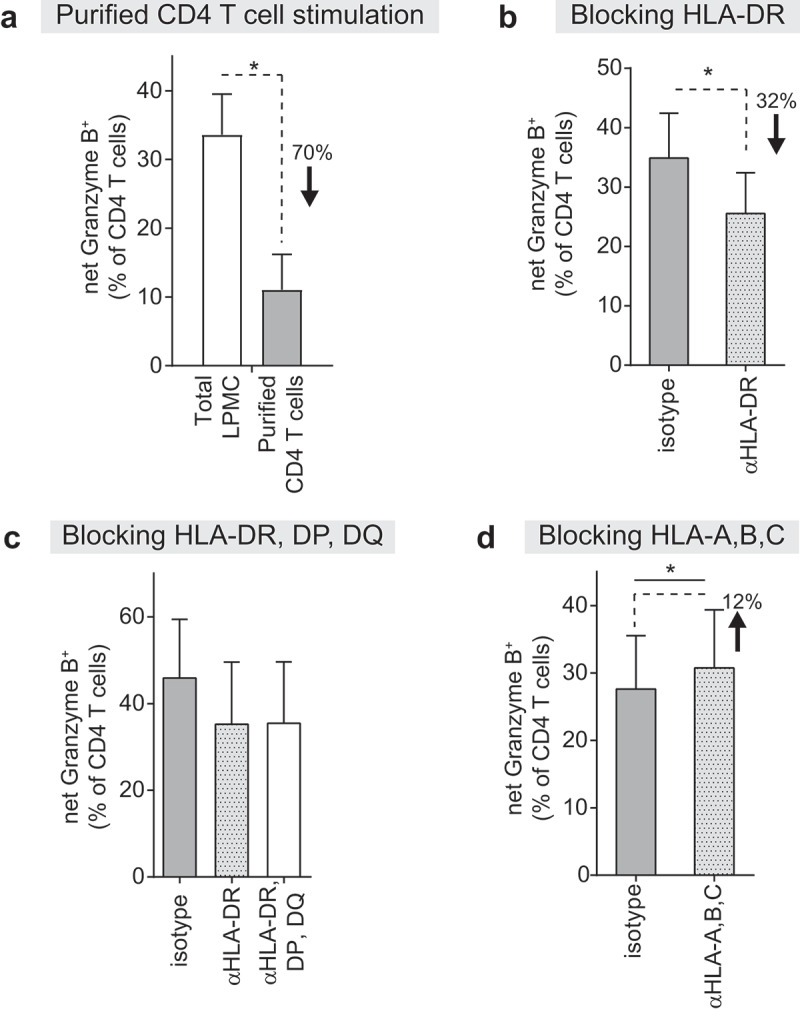
**Granzyme B expression in lamina propria CD4 T cells in response to *E. coli* primarily occurs in an indirect manner and is mediated, in part, by HLA-DR**. (a) Donor-matched total lamina propria mononuclear cells (LPMC) or purified lamina propria (LP) CD4 T cells (*N* = 4) were exposed *in vitro* to *E. coli* (10 μg/ml) for 4 days. (b-d) LPMC were pre-treated (30 mins) with 10 μg/ml of (b) anti-HLA-DR (αHLA-DR) (*N* = 7), (c) αHLA-DR, DP, DQ (*N* = 3) or (d) αHLA-A,B,C (*N* = 4) antibodies or matched isotype controls and cultured with and without *E. coli* (10 μg/ml) for 4 days. Values are shown as percentage of granzyme B (GZB)^+^ LP CD4 T cells in response to stimulation minus percentages detected in unstimulated conditions (net). Bars indicate mean+SEM. (%) indicates **(a)** average decrease in percentage of GZB^+^ LP CD4 T cells in purified CD4 T cell cultures versus total LPMC or **(b-d)** average percentage change in GZB^+^ LP CD4 T cells in conditions with blocking antibodies versus isotype control condition. Statistical analysis: Paired *t*-test; *P < .05.

Exposure of purified LP CD4 T cells to *E. coli* resulted in significantly lower percentages of GZB^+^ LP CD4 T cells relative to GZB^+^ LP CD4 T cells within similarly stimulated autologous LPMCs (Figure 5a). We next determined if GZB expression was dependent on the MHC. The presence of an HLA-DR (MHC Class II) blocking antibody significantly decreased the percentage of GZB^+^ LP CD4 T cells in response to *E. coli* stimulation of LPMC by an average of 32% versus no blocking antibody (Figure 5b). A strong correlation between GZB expression and proliferation of LP CD4 T cells following the HLA-DR blockade suggested the decreased percentage of GZB^+^ LP CD4 T cells in the presence of HLA-DR blocking was related to lower levels of proliferation (Figure S10). HLA-DP and HLA-DQ blockade did not further decrease GZB expression relative to HLA-DR blocking alone (Figure 5c). LPMC cultured with *E. coli* and HLA-A,B,C (MHC Class I) blocking antibodies resulted in marginally higher percentages of GZB^+^ LP CD4 T cells (Figure 5d).

### GZB^+^ LP CD4 T cells co-express proteins indicative of cytolytic capability

**Figure 6.**
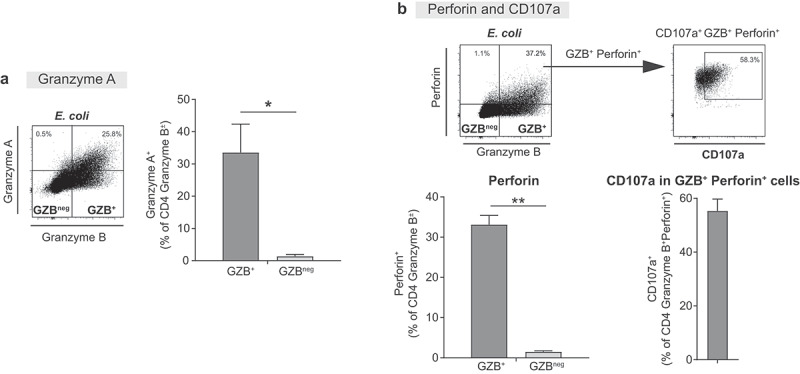
**Granzyme B^+^ lamina propria CD4 T cells co-express proteins indicative of cytolytic capacity**. Lamina propria mononuclear cells (LPMC) were exposed *in vitro* to *E. coli* (10 μg/ml) for 4 days. Expression of granzyme B (GZB^+^) in LP CD4 T cells was measured in conjunction with (a) Granzyme A (GZA) (*N* = 4) or (b) Perforin and CD107a (*N* = 3). GZB, GZA and perforin expression levels were established using a matched isotype control. %’s in representative flow plots reflect **(a)** GZA or **(b)** perforin in GZB^+^ LP CD4 T cells and those lacking GZB (GZB^neg^). Bar graphs represent summary values (mean+SEM) and compare expression of **(a)** GZA or **(b)** perforin in GZB^+^ or GZB^neg^ LP CD4 T cells. Fluorescently-labeled CD107a was added during the last 16 hours of culture and the unstimulated condition used to establish CD107a expression. A representative flow plot illustrating CD107a expression in GZB^+^ Perforin^+^ LP CD4 T cells is shown and values summarized as bar graphs (mean+SEM). Statistical analysis: Paired *t*-test; *P < .05, **P < .01.

We next measured GZB and co-expression of other molecules associated with cytolytic potential including GZA, perforin and CD107a (Lysosome-associated membrane glycoprotein-1, LAMP-1), a marker of degranulation (Figure 6). On average, 33.4 ± 8.8% of GZB^+^ LP CD4 T cells co-expressed GZA following *E. coli* lysate exposure (Figure 6a). Of note, almost all GZA-expressing cells co-expressed GZB (85.2 ± 6.0%). Perforin was expressed in 33.1 ± 2.3% of GZB^+^. Of GZB^+^Perforin^+^ LP CD4 T cells, 55.3 ± 4.5% expressed CD107a in response to *E. coli* lysate stimulation.

### GZB^+^ LP CD4 T cells expanded with *E. coli* lysate are enriched for cells capable of co-expressing IL-17 and IFNγ

**Figure 7.**
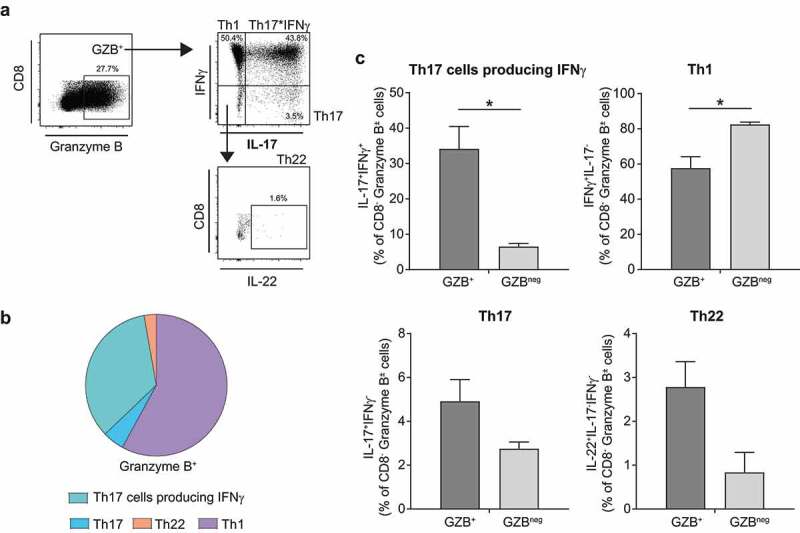
**Granzyme B-expressing lamina propria CD4 T cells induced in response to *E. coli* stimulation *in vitro* preferentially co-express IL-17 and IFNγ**. Lamina propria mononuclear cells (LPMC, *N* = 4) were exposed *in vitro* to *E. coli* (10 μg/ml) for 4 days. (a) Representative flow plots illustrating percentages of granzyme B (GZB)^+^ LP CD4 T cells co-expressing IFNγ only (IFNγ^+^IL-17^−^; T helper (Th) 1 cells), both IL-17 and IFNγ (IL-17^+^IFNγ^+^; Th17*IFNγ cells), IL-17 only (Th17 cells) or IL-22 only (IL-22^+^IFNγ^−^IL-17^−^; Th22 cells) following stimulation with *E. coli*. Due to downregulation of CD4 following short-term mitogenic stimulation, LP CD4 T cells were identified as CD8^−^ T cells. Matched isotype controls were used to establish GZB and cytokine expression. (b) Pie chart illustrating the average percentage of GZB^+^ LP CD4 T cells that were Th17*IFNγ^+^, Th17, Th22 or Th1 cells as a proportion of total GZB^+^ cells. (c) Bar graphs (mean+SEM) directly comparing percentages of Th cell subsets in GZB^+^ cells versus those lacking GZB (GZB^neg^). *N* = 4 for all except Th22 where *N* = 3. Statistical analysis: Paired *t*-test; *P < .05.

To evaluate the potential Th cytokine profile of *in vitro*-expanded GZB^+^ LP CD4 T cells, LPMC were cultured with *E. coli* for 4 days and exposed to mitogenic stimulation during the last 4 hours of culture. Flow cytometry was used to determine the percentage of GZB^+^ LP CD4 T cells capable of expressing IFNγ only (Th1 cells), IL-17 only (Th17 cells), both IL-17 and IFNγ (Th17*IFNγ cells) or IL-22 only (Th22 cells) (Figure 7a). *E. coli*-expanded GZB^+^ cells primarily co-expressed IFNγ only or both IL-17 and IFNγ (Figure 7b). GZB^+^ LP CD4 T cells contained a significantly higher fraction of Th17*IFNγ cells versus GZB^neg^ cells (Figure 7c). The fraction of Th1 cells within GZB^+^ LP CD4 T cells was significantly lower than Th1 cells in GZB^neg^ LP CD4 T cells. In general, low percentages (average <5%) of Th17 and Th22 cells were observed in GZB^+^ (and GZB^neg^) cells in response to *E. coli*.

### Rapid induction of GZB in response to *E. coli* lysate exposure is mediated by IL-2 and increased expression of IL-2Rα

**Figure 8.**
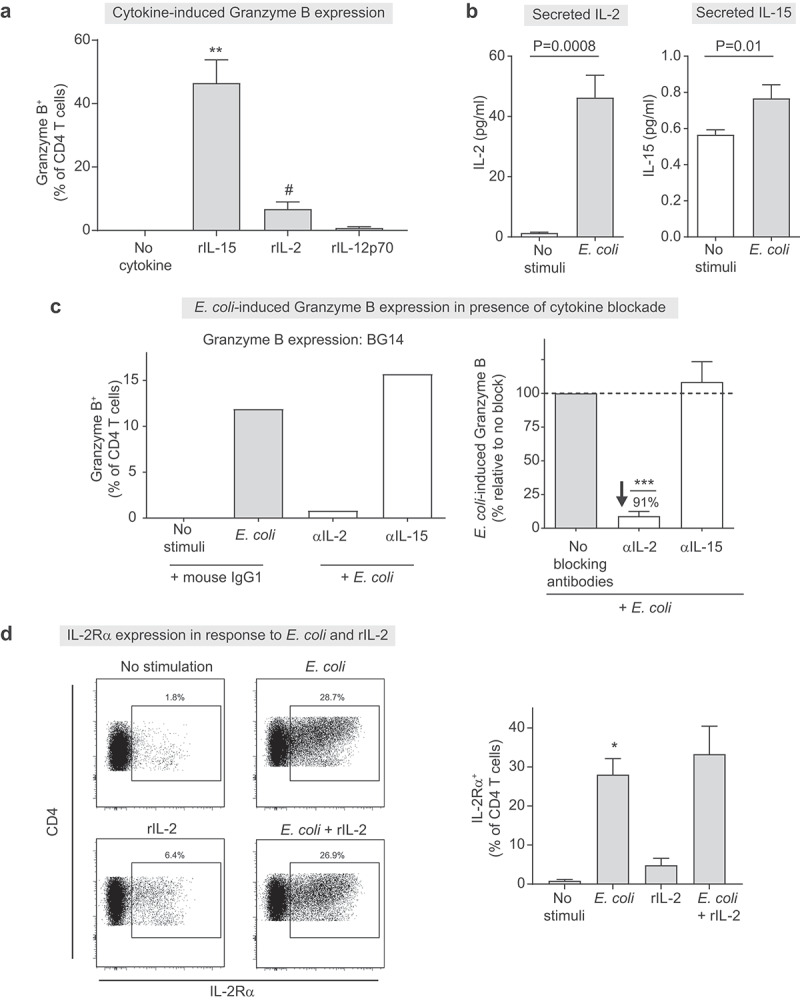
**Induction of granzyme B in lamina propria CD4 T cells following exposure of LPMC to *E. coli* is mediated by IL-2 and increased expression of IL-2Rα**. (a) Lamina propria mononuclear cells (LPMC, *N* = 6) were exposed *in vitro* to recombinant IL-15 (rIL-15; 50 ng/ml), rIL-12 (10 ng/ml), rIL-12p70 (50 ng/ml) or left untreated (no cytokine) for 48 hours prior to measurement of granzyme B (GZB) in LP CD4 T cells. Values are shown as the percentage of GZB^+^ LP CD4 T cells with isotype values removed. (b) LPMC (*N* = 7) were exposed to *E. coli* (10 μg/ml) or no exogenous stimulation for 48 hours and levels of secreted IL-2 and IL-15 measured in culture supernatants. (c) LPMC (*N* = 4) were pre-treated with 10 μg/ml blocking antibodies directed against IL-2 (αIL-2) or αIL-15 or with matched isotype controls and cultured with or without *E. coli* (10 μg/ml) for 48 hours. GZB expression in a representative sample is shown (left panel). Summary data (right panel) is shown as the percentage of GZB^+^ LP CD4 T cells in the presence of blocking antibodies relative to no blocking. (%) represents the mean percentage decrease in presence of αIL-2 relative to no blocking. (d) LPMC (*N* = 4) were exposed *in vitro* to *E. coli*, rIL-2, *E. coli* + rIL-2 or left untreated for 48 hours and IL-2 Rα expression measured. Left panel: representative flow plots illustrating percentages of IL-2 Rα^+^ LP CD4 T cells with expression established using the unstimulated condition. Right panel: Summary data is shown as the percentage of IL-2 R^+^ LP CD4 T cells. Bars indicate mean+SEM. Statistical analysis: Repeated Measures ANOVA relative to **(a)** no recombinant cytokine or **(c)** no cytokine blocking (normalized values), **(b)** Paired t-test, **(d)** analyzed by fitting a mixed model due to a missing value (*E. coli* + rIL-2). *P = .02, **P < .01, ***P < .001, ^#^*P* = .07.

Cytokines such as IL-2, IL-15 and IL-12p70 have been implicated as important drivers of murine and human cytotoxic cells.^46–50^ Therefore, to understand the mechanistic pathways driving GZB expression in response to bacterial stimulation, we next determined the ability of these cytokines (in the absence of bacteria) to drive the rapid induction of GZB in human LP CD4 T cells. LPMC were exposed to recombinant IL-2, (rIL-2), rIL-15 and rIL-12p70 and GZB expression in LP CD4 T cells evaluated after 48 hours (Figure 8a). Addition of rIL-15 to LPMC induced the greatest GZB expression in LP CD4 T cells, while rIL-2 also increased percentages of GZB^+^ LP CD4 T cells but to a lesser degree. Conversely, exposure of LPMC to rIL-12p70 induced minimal GZB^+^ LP CD4 T cells.

Given the ability of rIL-15 and rIL-2 to induce GZB expression in unstimulated LPMC, we next dissected the role of these cytokines in *E. coli*-induced GZB expression. Levels of secreted IL-2 and IL-15 significantly increased following *E. coli* exposure of LPMC, although the levels of IL-15 were generally low (<1 pg/ml) (Figure 8b). LPMC were next cultured with *E. coli* in the presence of antibodies directed against IL-2 and IL-15 (Figure 8c). Neutralizing IL-2 in the presence of *E. coli* significantly reduced GZB expression in LP CD4 T cells by an average of 91%. Conversely, neutralizing IL-15 had minimal effect (Figure 8c) even though the dose used effectively inhibited rIL-15-induced GZB expression (Figure S11). Given our observation that the RA of *Prevotella* species was predictive of frequencies of LP GZB^+^ CD4 T cells *in vivo*, we further investigated if IL-2 was critical in driving GZB expression in response to *P. stercorea*. In keeping with our published observations,^15^ *in vitro* exposure of LPMC (*N* = 4) to *P. stercorea* induced GZB expression in LP CD4 T cells within 48 hours (Figure S12a). Critically, blocking IL-2 resulted in an average decrease in GZB expression of 80% and in 3 of the 4 donor LPMC samples this decrease was greater than 90% (Figure S12b). Thus, IL-2 was also important for the induction of GZB in response to *P. stercorea*, the RA of which is predicative of higher colon LP GZB^+^ CD4 T cells *in vivo* (Table 2).

To further explore the observation that IL-2 was required for bacteria-mediated GZB (Figure 8c), but was less efficient at inducing expression when added in the absence of bacteria (Figure 8a), we next determined if this was related to the regulation of the IL-2Rα subunit (CD25). Exposure of LPMC to *E. coli* significantly increased IL-2Rα expression on LP CD4 T whereas the addition of rIL-2 to unstimulated LPMC only marginally increased GZB (Figure 8d). No reduction in IL-2Rα expression was noted with the combination (*E. coli* + rIL-2) demonstrating that rIL-2 did not block the ability to measure IL-2Rα expression.

## Discussion

HIV-1 infection is associated with epithelial barrier dysfunction that results in microbial translocation into the underlying lamina propria and systemic circulation.^2,4^ Our previous study modeling the interactions between the microbiome and gut lymphocytes *in vitro* led to a surprising discovery: enteric bacteria readily upregulated the cytotoxic marker GZB in gut CD4 T cells.^15^ However, to date, it remained unknown if these findings were just an artifact of cell culture. In this study, we readily detected and observed higher frequencies of GZB-expressing CD4 T cells in the colon of untreated, chronically infected PWH. These findings confirm that GZB^+^ CD4 T cells are a characteristic feature of HIV-1 infection in the gastrointestinal tract *in vivo*. We show that multiple enteric bacteria could induce GZB in gut CD4 T cells *in vitro*, but *in vivo*, the accumulation of colon GZB^+^ CD4 T cells positively associated with gut and systemic T cell activation and with the relative abundance of mucosa-associated *Prevotella* species. *Prevotella* species are commensal enteric bacteria abundant in PWH (likely related to sexual practices; i.e., receptive anal intercourse), and *Prevotella* enrichment is associated with alterations in gut homeostasis, including mucosal inflammation and T cell and mDC activation.^7,36,37,51–57^ Thus, our current study suggests a potential host–microbiome relationship that may shape the nature of gut mucosal CD4 T cells in PWH, especially those with abundant *Prevotella* species.

In addition to the gut, we also observed increased GZB^+^ CD4 T cells in the peripheral blood of PWH. The frequency of blood GZB^+^ CD4 T cells did not correlate with *Prevotella* species abundance. Instead, we noted a positive association between frequencies of blood effector memory GZB^+^ CD4 T cells and colonic GZB^+^ CD4 T cells in PWH. Human gut CD4 T cells are mostly composed of effector memory cells,^44^ and studies in mice demonstrated that tissue-resident CD8^+^ T cells can contribute to the circulating memory T cell pool following activation.^45^ Thus, the HIV-1-associated increase in blood GZB^+^ CD4 T cells may reflect recently activated gut GZB^+^ CD4 T cells migrating into the systemic circulation rather than differentiation of these cells in peripheral blood. Indeed, we observed that commensal bacteria-driven GZB induction in CD4 T cells *in vitro* was more robust in the gut versus blood, suggesting gut CD4 T cells are more prone to upregulate GZB in response to bacteria. This may be reflective of their residency and close proximity to gut microbiota and/or related to the fact that bacteria-reactive T cells are more frequent in gut tissue versus the blood.^10^

Major mechanistic questions remain on how gut CD4 T cells upregulate GZB expression following microbial exposure. While GZB induction was associated with T cell activation *in vivo*, not all GZB^+^ CD4 T cells induced by bacteria *in vitro* expressed T cell activation markers. Although GZB expression was most pronounced in Th17 cells co-producing IFNγ, it was also detected in Th1 and Th17 cells. By contrast, our *in vitro* studies demonstrated that the rapid induction of GZB was dependent on IL-2 with GZB expression likely further enhanced by bacteria-associated increases in IL-2Rα, the critical subunit of the high-affinity IL-2R.^58^ Of note, previous studies demonstrated that GZB expression was IL-2 dependent in murine CD4 T cells^46,49,50,59,60^ and in human blood Th1 and Th17 cells.^34^ High IL-2Rα expression was necessary for maximal GZB expression *in vivo* in response to Influenza A virus infection.^50^ We also described that GZB expression in LP CD4 T cells in response to bacterial stimulation of LPMC was not primarily an intrinsic feature of LP CD4 T cells. The expansion of GZB-expressing LP CD4 T cells in response to commensal *E. coli* was in part dependent on MHC Class II, as we had previously noted for mDC-dependent bacteria-induced proliferation of total CD4 T cells.^11,61^ Notably, our *in vivo* observations indicated positive associations between the frequencies of colon GZB^+^ CD4 T cells and CD11c^+^ mDCs. We therefore speculate that mDCs may drive GZB expression in gut CD4 T cells of PWH. Indeed, murine DCs rapidly produced IL-2 in response to multiple Toll-like receptor (TLR) ligands derived from both Gram-negative (e.g. LPS) and Gram-positive (e.g. LTA) bacteria and in response to *E. coli*,^62,63^ in keeping with our observations that both Gram-negative and Gram-positive enteric bacteria induced GZB expression in LP CD4 T cells. Human monocyte-derived DCs were also capable of producing IL-2, although only when derived in the presence of IL-15 and stimulated with CD40L, suggesting a requirement for T cell contact. Interestingly, IL-15 did not appear to play a critical role in bacteria-mediated induction of GZB in LP CD4 T cells *in vitro*. Given the retrospective nature of our current clinical study, we were unable to address the role of IL-2 in driving GZB expression in LP CD4 T cells in PWH. Both the source and regulation of IL-2, as well as the mechanisms by which it induces GZB expression in human gut LP CD4 T cells, requires further exploration.

The reason(s) for why gut CD4 T cells appeared to be primed for rapid GZB expression following microbial exposure remains unknown. Numerous studies showed that the presence of HIV-1-specific CD4 CTLs in the peripheral blood of PWH and that these cells may contribute to anti-viral immunity.^21–33^ By contrast, the lack of associations between frequencies of colon GZB^+^ CD4 T cells and either tissue or plasma HIV-1 RNA levels in PWH, and the fact that HIV-1 kills infected CD4 T cells, would suggest that the majority of GZB^+^ LP CD4 T cells in PWH may not be HIV-1 specific. Our *in vitro* studies did demonstrate that a considerable fraction of microbe-expanded GZB^+^ LP CD4 T cells expressed canonical cytolytic and degranulation markers. Thus, a fraction of the accumulated gut GZB^+^ CD4 T cells observed in untreated, chronically infected PWH may have cytolytic ability directed against cells harboring bacteria. If so, we predict that the activity of these CD4 CTLs in the gut may sustain gut barrier dysfunction and exacerbate mucosal inflammation. Additional studies will be required to dissect the targets and anti-bacterial cytolytic mechanisms of these LP CD4 CTLs. Moreover, it would be important to determine if these CD4 CTLs persist in the setting of ART, as ART-treated PWH still display features of dysbiosis and microbial translocation.^4,5^

Of note, a large fraction (average 67%) of bacteria-stimulated LP CD4 T cells expressed GZB in the absence of perforin. This finding raises the possibility that GZB expression may not completely equate to cytolytic killing. In recent years, a number of studies have investigated the non-cytolytic role of GZB. Specifically, extracellular GZB expression was linked to impaired wound healing,^64^ epithelial barrier disruption,^34,65^ induction of IL-1α and IL-18,^66,67^ and enhancing LPS-induced inflammatory cytokine production by APC.^20,68^ Intriguingly, GZB secretion in activated memory blood CD4 T cells was less strictly regulated than that of memory CD8 T cells, suggesting that CD4 T cells may be a major source of extracellular GZB.^69^ Thus, the non-cytolytic, pro-inflammatory properties of bacteria-induced GZB^+^ LP CD4 T cells may be yet another driver of pathogenic outcomes *in vivo*.

There are several limitations to our study. First, enumeration of GZB^+^ T cells was conducted using archived samples available from a small number of study participants, imbalanced for sexual practice which limits our ability to attribute the higher frequency of LP GZB^+^ CD4 T cells directly to HIV itself. A skewed distribution for some of the readouts may affect the fitting of the linear model. Furthermore, a larger study would allow for more complex modeling and also confirm the lack of significant associations truly reflected no relationship between the predictor and LP GZB^+^ CD4 T cell frequencies, rather than low statistical power for detecting relationships. Analysis of GZB expression *in vivo* was limited to histological examination of colon tissue that restricted our options for immunophenotypic analysis. Additional mechanistic studies are required to comprehensively evaluate the phenotype and function of microbe-expanded GZB-expressing LP CD4 T cells in the setting of HIV infection including determining whether the higher frequencies of LP CD4 T cells detected in PWH despite overall CD4 T cell depletion is related to a GZB-associated protective phenotype. The *in vitro* model used in this current study reflects the ability of bacterial antigens to induce GZB expression in human LP CD4 T cells. We recently demonstrated the bacterial metabolite butyrate, a short-chain fatty acid produced by bacteria following fermentation of non-digestible dietary fiber, reduced TCR-mediated gut CD4 T cell activation, proliferation, and inflammatory cytokine production *in vitro*.^70^ However, butyrate has also been shown to enhance IFNγ and GZB expression in murine CD8 CTLs.^71^ Therefore, given the altered gut microbiome of PWH typically reflects lower abundances of bacteria associated with the production of immune-modulating metabolites including butyrate,^5–7,43^ it would be critical to investigate how bacteria-associated metabolites modulate bacteria-induced GZB expression in human gut LP CD4 T cells.

In summary, this exploratory study provides evidence that GZB^+^ CD4 T cells may accumulate in colon tissue of untreated, chronically infected PWH. GZB was rapidly induced by diverse enteric bacteria primarily in gut but not blood or tonsil CD4 T cells, and this induction was dependent on IL-2 and in part, MHC Class II. Given the potential of these cells to cause inflammation via cytolytic and non-cytolytic mechanisms, these GZB^+^ CD4 T cells may be a critical factor in driving and sustaining gut mucosal HIV-1 pathogenesis, especially in the setting of epithelial barrier disruption, microbial translocation and dysbiosis. Further studies on the ontogeny, heterogeneity and function of these CD4 T cells in the gut may inform strategies to curb inflammation-associated comorbidities in PWH, as well as other diseases in which dysbiosis and microbial translocation contribute to altered gut immunity, such as in inflammatory bowel diseases.^72^

## Materials and Methods

### Clinical study participants and study design

Archived formalin-fixed, paraffin-embedded (FFPE) colonic tissue and PBMC were obtained from 10 chronically-infected PWH who were not receiving ART and 10 HIV-1 uninfected adult control participants enrolled in a completed IRB-approved cross-sectional study at the University of Colorado Anschutz Medical Campus.^36,37,43,73,74^ All study participants voluntarily gave written, informed consent. Inclusion and exclusion criteria and the collection, storage and processing of colon biopsies, PBMCs, plasma and serum have been extensively detailed in previous publications.^36,37,74^ The study participants were selected from a larger, previously completed clinical study that included 17 PWH and 14 uninfected controls with previously acquired gut bacterial microbiome datasets^36,37^ and an adequate amount of FFPE tissue to permit histological assessment of at least 1.0 mm^2^ of total tissue area (see below). PWH were then retrospectively selected for this current study based on blood CD4 counts ≥400 cells/μl to enrich for participants with an adequate number of colonic CD4 T cells to permit accurate histological enumeration of GZB^+^ cells. The final cohort of PWH (*N* = 10) was then selected based on the highest ranking of (1) colonic tissue HIV-1 RNA levels (HIV-1 replication), (2) systemic IL-6 levels (inflammation) and (3) systemic LPS levels (microbial translocation). Uninfected controls with blood CD4 counts ≥400 cells/μl were selected to balance both age and sex of the PWH cohort (Table S1).

### Histological staining and analysis of GZB expression in human colonic biopsies

Archiving of FFPE colon biopsy tissue occurred between November 2011 and November 2012. For all staining protocols, tissue sections were freshly cut prior to histology and any tissue area/sections that were poorly stained (e.g. weak signal) were excluded from the analyses. Biopsy tissue was cut into 4 μm thick sections (CU AMC Tissue Biobanking and Histology Shared Resource Center). Three sequential sections from each of three biopsies per study participant were analyzed. Multispectral imaging was performed by the CU-AMC Human Immune Monitoring Shared Resource (HIMSR) using the Vectra 3.0 Automated Quantitative Pathology Imaging System (Akoya Biosciences, Inc.). Slides were sequentially stained for human CD3 (LN10, Leica Biosystems), EPCAM (MOC31, Leica Biosystems), Granzyme B (GrB-7, Invitrogen) and CD8 (C8/144B, Agilent-Dako) using the Leica Bond RX autostainer (Leica Biosystems). Slides were deparaffinized, heat treated in ER2 (epitope retrieval solution 2) antigen retrieval buffer (Leica), blocked in antibody diluent (Akoya Biosciences, Inc) and incubated with the primary Ab, followed by horseradish peroxidase (HRP)-conjugated secondary antibody polymer (HRP, Akoya Biosciences) and HRP-reactive OPAL fluorescent reagents (Opal 650, 620, 540, 690); Akoya Biosciences). Slides were washed between staining steps with Bond Wash (Leica) and stripped between each round of staining with heat treatment in appropriate antigen retrieval buffer. After the final staining round, the slides were heat-treated in ER1 antigen retrieval buffer, stained with spectral DAPI (Akoya Biosciences), and coverslipped with Prolong Diamond mounting media (Thermo-Fisher Scientific, Waltham, MA). Whole slide scans were collected using the 4x objective and up to 18 regions of interests identified using Phenochart 1.0 (Akoya Biosciences) for an additional scan at 20X. Scanned images were spectrally separated and prepared using inForm Tissue Finder (v.2.4, Akoya Biosciences). Images were blinded and Granzyme B^+^ CD3^+^ CD8^±^ T cells identified in the lamina propria using Image J Software (NIH). On average, a total area of 1.5 mm^2^ (range 1.0–1.8 mm^2^) was analyzed for Control participants and 1.6 mm^2^ (range 1.2–2.0 mm^2^) for PWH. Due to down-regulation of CD4 by HIV,^39^ CD4 T cells were identified as CD3^+^ CD8^neg^ and GZB-expressing CD8^neg^ T cells reported as percentage of total CD8^neg^ T cells and as number of GZB^+^ CD8^neg^ T cells per mm^2^ of tissue area. In a subset of study participants (*N* = 3 controls, *N* = 3 PWH), tissue sections were stained with γδ TCR (5A6.E9, Invitrogen; Opal 570) in addition to the previously detailed antibodies (CD3, Opal 650; CD8, Opal 690; EPCAM, Opal 620) and analyzed for frequencies of γδ TCR^+^ cells expressing GZB (average total area analyzed: controls 2.6 ± 0.6 mm^2^, PWH: 2.5 ± 0.2 mm^2^).

### Measurement of GZB expression in peripheral blood CD4 T cells

PBMC were isolated by standard Ficoll-Hypaque (GE Healthcare) density gradient centrifugation, cryopreserved and stored in liquid nitrogen as previously described.^36^ PBMC were thawed and surface and intracellular expression of various markers expressed by peripheral blood CD4 T cells was determined using multi-color flow cytometry. Specifically, standard flow cytometry staining protocols were used to determine expression of viable cells (Aqua Zombie Live/Dead Fixable cell dye, Biolegend), cell surface markers CD3 (BV605 CD3, clone UCHT1; Biolegend), CD4 (BUV395 CD4, clone SK2, BD Biosciences), CD8 (PE Dazzle, clone RPA-T8, Biolegend), CCR7 (APC-Cy7 CCR7, clone G043H7, Biolegend) and CD45RO (PE CD45RO, clone UCHL1, Biolegend) and intracellular expression of GZB (Pacific Blue GZB, clone GB11, Biolegend) in Medium A Fixation buffer and Medium B Permeabilization buffer (both Life Technologies, Thermo Fisher Scientific) as previously detailed.^8,9,12^ Fluorochrome matched isotype controls were used to establish naïve (CCR7^+^CD45RO^−^), central memory (CM, CCR7^+^CD45RO^+^), effector memory (EM, CCR7^−^CD45RO^+^), and terminally differentiated effector memory (TD, CCR7^−^CD45RO^−^) CD4 T cells and intracellular GZB expression (mouse IgG1). Data was acquired on an LSRII flow cytometer (BD Biosciences) with routine quality control by Cytometer Setup and Tracking feature within the BD FACSDiva software version 6.1.2 (BD Biosciences) performed routinely. Flow cytometry data analysis was performed using FlowJo v10.0. The gating strategy for a representative sample is shown in Figure S5. In one control study participant, the percentage of GZB-expressing blood CD4 T cells was identified as an outlier (Grubbs Outlier Test; GraphPad Software) and all associated PBMC values were excluded from the analyses.

### Measurement of serum GZA and GZB levels

Serum levels of GZA and GZB were measured using commercially available ELISAs (Invitrogen) following manufacturers protocols. Limits of sensitivity were 0.73 pg/ml and 0.2 pg/ml for Granzyme A and Granzyme B respectively.

### Previously obtained immunological, virological and microbial measurements

Methods for measuring the immunological, virological and microbial variables are extensively detailed elsewhere^36,37,74^ and briefly detailed in supplementary methods.

### Statistical analysis for clinical study

Participant demographics and characteristics are reported as median (minimum, maximum) for continuous measures and as frequency (percent) for categorical measures (Table S1). Mann-Whitney U tests were used to compare medians of continuous characteristics between the control and PWH groups. Fisher’s exact tests were used to compare categorical characteristics between the control and PWH groups. Frequencies of colonic CD8^neg^ T cells (reported as percentage of total CD3^−^ T cells and as number/mm^2^) and frequencies of GZB^+^ CD8^neg^ T cells (reported as percentage of CD8^neg^ T cells and number of GZB^+^ CD8^neg^ T cells/mm^2^) were compared between control and PWH groups using two-sample Mann-Whitney U tests. Linear models were used to evaluate the association between study participant variables (see above; predictors) and GZB-expressing CD8^neg^ T cells (outcome) in controls versus PWH when adjusting for sex, age, and HIV status. *P*-values were adjusted using Benjamini–Hochberg False Discovery Rate (FDR). Variables were only included if measured in at least 18 of the 20 participants. Outlier tests were run on all GZB expression values using Grubbs Outlier Tests, GraphPad Prism version 8 and 9 for Windows (GraphPad Software).

## Collection and isolation of human LPMC, TMC and PBMC for *in vitro* studies

*LPMC*: Jejunum samples (*N* = 31) were acquired from patients undergoing elective abdominal surgery and were designated discarded tissue from macroscopically normal sites. Samples from patients with a history of Inflammatory Bowel Disease, HIV-1 infection, treatment with immunosuppressive drugs, or recent chemotherapy (within 8 weeks) were excluded from the study. LPMC were isolated from tissue samples in a two step-procedure to remove epithelial cells followed by collagenase-digestion to release LPMC as previously described.^8,9,11,12,38^ LPMC were cryopreserved and stored in liquid nitrogen. All patients undergoing surgery signed a release form to allow unrestricted use of discarded tissue for research purposes. Protected patient information was de-identified to the laboratory investigators. Research associated with the use of LPMC was reviewed by the Colorado Multiple Institutional Review Board (COMIRB) at UC-AMC and deemed Not Human Subject Research as defined by their polices in accordance with OHRP and FDA regulations. Each patient who provided a tissue specimen was considered a single sample and a minimum of 3 samples was used for each assay.

*TMC*: Human tonsillar tissue samples (*N* = 5) were obtained from pediatric patients at low risk for HIV-1 infection undergoing routine tonsillectomies at Colorado Children’s Hospital, Aurora, CO. TMC were isolated by mincing, straining through a 70uM nylon filter followed by RBC lysis and additional straining and washing with DPBS. Isolated TMC were cryopreserved and stored in liquid nitrogen. Protected patient information was de-identified to the laboratory investigators and research associated with the use of TMC was reviewed by COMIRB and deemed Not Human Subject Research.

*PBMC*: Blood samples were obtained from healthy adults (*N* = 5) and PBMC isolated using standard Ficoll-Hypaque density gradient centrifugation, cryopreserved and stored in liquid nitrogen as previously detailed.^75–77^ All the study subjects participated voluntarily and gave written, informed consent. This study was approved by COMIRB.

## *In vitro* stimulation of LPMC, TMC or PBMC with whole bacteria, bacterial lysates or TCR-stimulatory beads

*In vitro cell culture*: LPMC, TMC or PBMC were thawed and cultured in RPMI with 10% human AB serum (Gemini Bioproducts), 1% Penicillin/Streptomycin/Glutamine (Life Technologies), and 500 μg/ml Zosyn (Piperacillin and Tazobactam) at 1.0 × 10^6^ cells/ml in a 48-well plate (unless noted) at 37°C, 5% CO_2_ for 1–4 days.

*Stimulation with whole bacteria or bacterial lysates: Escherichia coli* (ATCC 25922), *Salmonella typhimurium* (ATCC 35986), *Acinetobacter junii* (ATCC 17908), *Bifidobacterium longum subsp. infantis* (*B. infantis*; ATCC 15697), *Bacteroides thetaiotaomicron* (ATCC 29148), *Ruminococcus bromii* (ATCC 27255) and *Prevotella stercorea* (DSM No. 18206, DSMZ, Braunschweig, Germany) were expanded in appropriate aerobic or anaerobic media as previously detailed.^12,78^ Bacterial cell counts were determined using the BD Cell Viability Kit (BD Bioscience) and all stocks were stored as single use aliquots at −80°C. Bacterial lysates were prepared from the same whole-cell bacterial stocks via bead beating and heating, and the protein concentration was determined using Pierce BCA assay (Thermo-Fisher Scientific) prior to storage at −80°C as previously described.^8^ LPMC were stimulated with whole bacteria at a ratio of 2.5 bacteria to 1 LPMC. Bacterial lysates were added to LPMC, TMC or PBMC cultures at 10 μg/ml.

*Stimulation with TCR-stimulatory beads*: T cell activation was achieved using beads directed against CD3, CD2 and CD28 (T cell Activation/Expansion Kit, Miltenyi Biotec) at a ratio of 1 bead to 25 LPMC/TMC or 1 bead to 2 PBMC.

## T cell proliferation and MHC Class II (HLA-DR, DP, DQ) or MHC Class I (HLA-ABC) blocking assays

LPMC were pre-labeled with 1 μM CellTrace CFSE (Invitrogen) per manufacturer’s instructions. CFSE-LPMC (1x10^6^ cells/ml) were pre-treated with antibodies directed against HLA-DR, HLA-ABC (Leaf; BioLegend) or HLA-DR, DP, DQ (BD Biosciences) (all at 10 μg/ml) or matched isotype controls for 30 mins at 37°C and 5% CO_2_ with gentle swirling after 15 mins. LPMC were plated in triplicate in 96 V-bottom plate and stimulated with or without *E. coli* lysate (10 μg/ml). . LPMC were cultured at 37°C with 5% CO_2_ for 4 days with additional blocking antibodies (or isotypes) added after 24 hours.

## Mitogenic stimulation of LPMC to determine frequencies of Th cells

LPMCs were stimulated with 25 ng/ml phorbol myristate acetate (PMA; Sigma-Aldrich) and 1 μg/ml ionomycin (Sigma-Aldrich) in the presence of 1 μg/ml Golgi Plug (Brefeldin A; BD Biosciences) for the last 4 hours of culture.

## Isolation and *in vitro* culture of purified LP CD4 T cells

LP CD4 T cells were isolated from the total LPMC using immunomagnetic negative selection (EasySep Human CD4^+^ T cell isolation kit; Stemcell Technologies) according to the manufacturer’s instructions. To control for differences in cell responses due to the isolation process itself, total LPMCs also underwent magnetic negative selection, but the EasySep separation buffer was used in place of the CD4 T cell Isolation Antibody Cocktail. On average LP CD4 T cells were enriched to 94.5 ± 0.8% (SEM) of viable CD45^+^ cells (*n* = 4). Total LPMC and purified LP CD4 T cells were cultured at 1.0 × 10^6^ cells/ml in the presence of *E. coli* lysates (10 μg/ml) or left unstimulated at 37°C, 5% CO_2_ for 4 days.

## Short-term *in vitro* exposure of LPMC to recombinant cytokines

LPMCs were cultured with or without recombinant IL-2 (10 ng/ml; rIL-2; Tonbo Biosciences), rIL-15 (50 ng/ml; R&D Systems) or rIL-12p70 (50 ng/ml; BioLegend) at 37°C with 5% CO_2_ for 2 days.

## Short-term *in vitro* exposure of LPMC to cytokine neutralizing antibodies

LPMC were pre-treated with antibodies directed against IL-2 or IL-15 (all 10 μg/ml; R & D Systems) or with matched isotype controls for 30 mins with gentle swirling after 15 mins. LPMC were cultured with or without *E. coli* or *P. stercorea* lysate (both 10 μg/ml) at 37°C with 5% CO_2_ for 2 days.

## Measurement of secreted cytokines

Levels of IL-2 and IL-15 in culture supernatants collected after 2 days of *in vitro* culture of LPMC in the presence or absence of *E. coli* lysates (10 μg/ml) were measured using a U-PLEX Assay and quantified on the QuickPlex SQ 120 Instrument according to manufacturer’s instructions (Mesoscale Discovery).

## Flow cytometry staining, acquisition, and analysis

Standard flow cytometry staining protocols to determine expression of cell surface markers (CD45, CD3, CD4, CD8, IL-2 receptor (IL-2R; CD25), proliferation (CFSE) and intracellular expression of granzymes, perforin, IFNγ, IL-17 and IL-22 using Medium A Fixation buffer and Medium B Permeabilization buffer (both Life Technologies) were followed as previously detailed.^8,9,12^ All antibodies and dyes are listed in Table S9. Data was acquired on an LSRII flow cytometer and flow cytometry data analysis performed using FlowJo v10.0 as detailed above.

To identify GZB-expressing LP CD4 T cells directly *ex vivo* (i.e. baseline) and following 1–2 days of *in vitro* culture, total CD4^+^ T cells were identified as CD3^+^ CD8^neg^ T cells within CD45^+^, viable (aqua dye-) lymphocytes (based on forward and side scatter properties) with doublets excluded based on forward-scatter-height versus forward-scatter-width properties. For identification of GZB-expressing LP CD4 T cells following 4 days of culture a similar gating strategy was employed, but without the inclusion of CD45. Due to down-regulation of CD4 following mitogenic stimulation, CD4 T cells were gated as CD3^+^ CD8^neg^ T cells when determining frequencies of cytokine-expressing GZB^+^ cells. Isotype controls were used to establish GZB, GZA, perforin, IFNγ, IL-17 and IL-22 expression. Proliferating LP CD4 T cells were enumerated as the percentage of CFSE^lo^ CD4 T cells with the unstimulated condition used to establish the CFSE gate. To measure CD107a expression by GZB^+^ perforin^+^ LP CD4 T cells following *E. coli* or TCR bead stimulation, fluorescently labeled CD107a was added during the last 16 hours of culture with an unstimulated condition used as the control to establish stimulation-specific CD107a expression. Specific gating strategies are shown in Figures 4 (GZB expression in LPMC, TMC and PBMC), Figure 6 (GZB^+^ and GZB^neg^ cells expressing GZA, perforin or CD107a), Figure 7 (GZB^+^ and GZB^neg^ cells co-expressing IFNγ, IL-17 or IL-22), Figure 8 (IL-2Rα expression), Figure S6 (baseline expression of GZB in CD4 and CD8 T cells), Figure S7 (GZB expression following bacterial stimulation), and Figure S9 (GZB expression in proliferated or activated LP CD4 T cells).

## Statistical analysis for *in vitro* studies

Statistical analysis and graphing were performed using GraphPad Prism version 8 for Windows (GraphPad Software, San Diego, CA, USA). Paired t-tests were used to compare LP CD4 versus CD8 T cells, stimulated versus unstimulated conditions, GZB^+^ LP CD4 T cells versus GZB^neg^ T cells, purified CD4 T cells versus total LPMC and blocking versus no blocking conditions. Repeated Measures ANOVA test was used for multiple comparisons. Each patient who provided a tissue specimen was considered a single sample for data analysis and the number of samples used in each assay are detailed in text and/or figure legends.

## Data Availability

Raw paired-end Illumina MiSeq reads were submitted to the NCBI Small Read Archive under BioProject accession number PRJNA227062 as previously reported.^36^ The data that support the findings of this study are available from the corresponding author [CCW], upon reasonable request.
